# Factors influencing childbirth intention and the moderating role of values regarding children among nursing students in Korea: a cross-sectional study

**DOI:** 10.4069/whn.2025.08.20

**Published:** 2025-09-30

**Authors:** Yunmi Kim, Seryeong Kim

**Affiliations:** College of Nursing, Jesus University, Jeonju, Korea

**Keywords:** Birth rate, Marriage, Nursing, Parturition, Pregnancy

## Abstract

**Purpose:**

This study aimed to identify factors influencing childbirth intention among nursing students, with particular focus on the relationship between marital attitude and childbirth intention, and whether values regarding children moderate this relationship.

**Methods:**

A descriptive correlational study was conducted with 205 unmarried third- and fourth-year nursing students from eight colleges in Jeollabuk-do, Korea. Participants completed an online survey on childbirth intention, marital attitude, and values regarding children. Data were analyzed using hierarchical multiple regression and moderation analysis (PROCESS Model 1).

**Results:**

Values regarding children, reported at a moderate level, were the strongest positive predictor of childbirth intention, which was slightly below moderate. Childbirth intention showed a positive correlation with values regarding children (r=.61, *p*<.001) and marital attitude (r=.15, *p*=.019). Among the marital attitude subdomains, conservative and active attitudes were positively associated, while a passive attitude was negatively associated, with childbirth intention. Childbirth intention was also positively related to economic satisfaction (r=0.19, *p*=.003) but not to major satisfaction (r=0.10, *p*=.078). Values regarding children were positively correlated with economic satisfaction (r=0.18, *p*=.004) and moderated the relationship between marital attitude and childbirth intention (B=.02, *p*=.04). Simple slope analysis revealed that marital attitude negatively affected childbirth intention only among students with low values regarding children (B=–.19, *p*=.029).

**Conclusion:**

Childbirth intention among nursing students is primarily shaped by values regarding children and marital attitudes. In particular, a low valuation of children amplifies the negative effect of marital attitudes.

## Introduction

South Korea (hereafter, Korea) recently reported its lowest-ever total fertility rate of 0.72 births per woman, making the declining willingness of young adults to have children a critical national concern [1]. The fertility rate has dropped sharply from 6.0 births per woman in 1960 and has remained well below the Organisation for Economic Co-operation and Development (OECD) average for the past four decades [2]. Furthermore, the average maternal age at childbirth has risen from 29.0 years in 2000 to 33.5 years in 2022, about 2.5 years higher than the OECD average [2]. The combination of delayed childbearing and persistently low fertility threatens the demographic structure and reproductive sustainability of the country, underscoring the urgent need to examine childbirth intentions among reproductive-aged young adults.

Nursing students in particular occupy a critical position for future family and reproductive health education, both as future healthcare professionals and as potential educators and role models in clinical and community settings [3]. However, previous studies have shown that nursing students often report lower levels of childbirth intention than peers in other disciplines, largely due to concerns about work-family conflict [3], financial difficulties, and insufficient social support [4]. Understanding their childbirth intentions is therefore essential not only for shaping national fertility policies but also for designing value-based life-planning education, as well as counseling and mentoring programs within nursing curricula.

Although nursing students receive systematic education in women’s health, maternal nursing, and pediatric nursing—disciplines that may encourage structured perspectives on marriage, childbirth, and parenting—studies in Korea indicate that they generally report lower levels of childbirth intention compared to students in other majors [3,5]. This appears to be associated with practical challenges, including balancing professional and family roles [3], financial barriers [5], and limited social support [5].

Research in Korea has consistently identified the value placed on children as one of the strongest predictors of childbirth intention among female university students [4] and nursing students [6]. A recent systematic review confirmed that stronger personal and societal values regarding children are consistently associated with higher fertility intentions [7]. Attitudes toward marriage also significantly influence childbirth intention. Specifically, passive marital attitudes tend to lower childbirth intention, whereas active and conservative attitudes are associated with increases [4]. However, most existing research has examined these factors as separate influences or simple correlations. There remains a lack of empirical evidence on how marital attitudes and child-related values interact in shaping childbirth intention, particularly whether values regarding children moderate this relationship [8]. In addition, while prior studies have examined nursing students broadly or focused primarily on female students without differentiating year of study, few have investigated third- and fourth-year students specifically. These students are engaged in maternity and pediatric nursing coursework and clinical placements, where they encounter childbirth and child-rearing scenarios firsthand [7,9]. At this stage, students are navigating a transitional period in which they refine career paths and make life-planning decisions. Their perspectives on marriage and childbearing may significantly influence not only their own futures but also their professional roles in family and reproductive health education.

Therefore, this study sought to investigate how marital attitudes and values regarding children influence childbirth intention among nursing students, with a particular focus on the moderating role of child-related values.

## Methods

**Ethics statement**

This study was approved by the Institutional Review Board of Jesus Hospital (No. 2024-04-014). Participation in the survey was considered as voluntary consent to the study. All responses were anonymous, no personally identifiable information was collected, and procedures followed the protocol approved by the Institutional Review Board.

### Study design

This descriptive correlational study employed a cross-sectional online survey to investigate factors influencing childbirth intention among nursing students in Korea. Reporting followed the STROBE (Strengthening the Reporting of Observational Studies in Epidemiology) guidelines (https://www.strobe-statement.org/).

### Participants

Participants were nursing students enrolled in Jeollabuk-do, Korea. Convenience sampling was used for recruitment. The inclusion criteria were: (1) unmarried status and age between 20 and 40 years; (2) enrollment as a third- or fourth-year student; and (3) completion of, or current engagement in, clinical practice in women’s health nursing or pediatric nursing. Exclusion criteria were: (1) currently receiving counseling or medication for psychiatric or mood disorders, and (2) being married, as the study focused on unmarried individuals of reproductive age who had not yet made personal decisions regarding marriage and childbirth. Third- and fourth-year students were selected because they are at an advanced stage of education, participating in theoretical and clinical coursework in women’s health and pediatric nursing. This level of training provides opportunities to experience and reflect on childbirth and child-rearing in clinical settings.

The required sample size was calculated using G*Power version 3.1.9.2 (Heinrich Heine University Düsseldorf, Düsseldorf, Germany) [10] for hierarchical multiple regression. With a significance level of 0.05, power of 0.95, and a medium effect size (f²=0.15), based on prior research indicating that predictors explained approximately 12% to 20% of the variance in childbirth intentions among nursing students [5,11], a minimum of 204 participants was required for 16 predictor variables. Data were collected from 205 students, and all responses were retained after screening for completeness and duplication. To prevent multiple submissions, Google Forms (Google LLC, Mountain View, CA, USA) was set to accept only one response per device, and no duplicate IP addresses were detected during data review. There were no missing values or dropouts, resulting in a valid response rate of 100%.

### Study variables and measures

The instruments used in this study were employed after obtaining permission via email from the original developers and translators of the tools.

#### Childbirth intention

Childbirth intention was assessed using an 11-item scale originally developed to measure fertility intentions among Korean young adults and university students [12]. The scale comprises four dimensions: personal (two items), familial (two items), economic (four items), and policy-related (three items). Items are rated on a 5-point Likert scale from 1 (“not at all”) to 5 (“very much”), with higher total scores (range, 11–55) reflecting stronger intentions to have children. Internal consistency was previously reported as Cronbach’s α=0.78 (personal), 0.69 (familial), 0.85 (economic), and 0.65 (policy-related) [12]. In this study, the overall Cronbach’s α was 0.79.

#### Marital attitude

Marital attitude was measured using a modified version [13] of a 20-item instrument originally designed for unmarried women and adolescents [14]. This tool assesses perceptions and values related to marriage and includes six subdomains: romantic (four items), passive (four items), conservative (three items), exclusive (three items), active (three items), and instrumental (three items). Each item is rated on a 5-point Likert scale (1, “not at all” to 5, “very much”), with higher total scores (range, 20–100) indicating stronger marital attitudes. Cronbach’s α for the total scale was 0.62 in a previous study using the modified version [14], with subscale reliabilities ranging from .55 to .62. In this study, the total scale reliability was .65, and the subscale reliabilities were: romantic (.60), passive (.66), conservative (.53), exclusive (.50), active (.40), and instrumental (.61).

#### Values regarding children

Values regarding children were assessed using an eight-item scale originally developed for Korean adults [15]. Items were rated on a 5-point Likert scale from 1 (“not at all”) to 5 (“very much”), with higher total scores (range, 8–40) indicating stronger values or positive perceptions regarding having children. The internal consistency reliability of the instrument was previously reported as .86 [15] and was .81 in this study.

#### General characteristics of the participants

General demographic information was collected on variables previously identified as predictors of childbirth intention: sex and age [5,11], satisfaction with nursing major and economic satisfaction [11,16], as well as religion, birth order, parents’ marital status, and cohabitation with parents [17]. Grade year and age were assessed using screening questions.

### Data collection

Data were collected from May 16 to July 31, 2024, using an anonymous Google Forms questionnaire. Recruitment flyers containing a description of the study’s purpose, inclusion and exclusion criteria, and voluntary nature were posted on bulletin boards and online platforms across eight nursing colleges in Jeollabuk-do, Korea. These eight institutions represented all eligible 4-year nursing colleges in the province offering comparable curricula and clinical training in women’s health and pediatric nursing. As such, the sampling approached a census of the target population. The flyer included a QR code linking to an electronic information sheet that explained confidentiality and the right to withdraw at any time. Only participants who selected “I consent” were able to proceed to the survey. Screening questions assessed eligibility regarding age, year in nursing school, and completion or current participation in clinical practice in women’s health or pediatric nursing. The survey required approximately 15 minutes to complete. After completion, participants received a mobile gift certificate worth 5,000 Korean won (approximately 4 US dollars) as a token of appreciation. 

### Data analysis

All statistical analyses were conducted using IBM SPSS for Windows, ver. 26.0 (IBM Corp., Armonk, NY, USA). Descriptive statistics were used to summarize participants’ general characteristics and the primary study variables, including marital attitude, values regarding children, and childbirth intention.

The independent t-test and one-way analysis of variance were performed to examine differences in childbirth intention across participants’ general characteristics. Pearson correlation coefficients were calculated to assess the relationships among the main variables.

To identify significant predictors of childbirth intention, hierarchical multiple regression analysis was carried out using the enter method. General covariates (sex, satisfaction with nursing major, and economic satisfaction) were entered in the first block, followed by the subdomains of marital attitude and values regarding children in subsequent blocks to assess their incremental explanatory power.

The moderating effect of values regarding children on the relationship between marital attitude and childbirth intention was tested using the PROCESS macro for SPSS (Model 1) developed by Hayes [18] and Hayes and Matthes [19]. When a significant interaction was found, simple slope analysis and the Johnson–Neyman technique were used to interpret the conditional effects of marital attitude on childbirth intention at three levels of child-related values: low (mean – 1 standard deviation [SD]), moderate (mean), and high (mean + 1 SD). All statistical tests were two-tailed, with significance set at *p*<.05.

## Results

### Differences in childbirth intention by general characteristics

Table 1 presents the differences in childbirth intention across participants’ general characteristics. Statistically significant differences were observed by sex, age, satisfaction with nursing major, and economic satisfaction. Male students reported significantly higher childbirth intention scores (mean±SD, 32.23±5.36) than female students (28.07±6.24) (t=4.37, *p*<.001). By age, participants aged 25 to 30 years showed the highest childbirth intention scores (31.42±5.05), with differences across groups reaching statistical significance (F=4.37, *p*=.014). Students who reported satisfaction with their nursing major had significantly higher scores (29.47±6.32) than those who were dissatisfied (23.13±6.29) (F=7.36, *p*<.001). Post hoc analysis using the Scheffé test indicated that both the satisfied and moderately satisfied groups scored higher in childbirth intention than the dissatisfied group. Similarly, students satisfied with their economic situation reported significantly higher childbirth intention (30.43±6.61) compared to dissatisfied students (27.00±6.44) (F=4.26, *p*=.015). No significant differences in childbirth intention were observed according to grade year, religion, birth order, parents’ marital status, or cohabitation with parents.

### Childbirth intention, marital attitude, and values regarding children

As shown in Table 2, participants reported a moderate level of childbirth intention (28.94±6.29) and a moderately positive level of values regarding children (26.31±5.12). These results indicate that while participants generally valued childbearing and child-rearing positively, their actual intention to have children was somewhat reserved. The mean marital attitude score was also at a moderately positive level (69.60±6.01), exceeding the scale midpoint (60 on a 20–100 scale). Subdomain scores were as follows: romantic attitudes (21.15±2.26), passive attitudes (19.35±4.01), conservative attitudes (7.86±2.19), exclusive attitudes (7.32±1.47), active attitudes (6.25±1.77), and instrumental attitudes (7.68±1.60). Effect size analysis revealed that passive attitudes (η²=.27, *p*<.001), active attitudes (η²=.25, *p*<.001), and values regarding children (η²=.45, *p*<.001) had strong and statistically significant effects on childbirth intention. Other subdomains—romantic attitudes (η²=.11, *p*=.019), conservative attitudes (η²=.12, *p*=.007), and exclusive attitudes (η²=.12, *p*=.001)—also showed significant but smaller effects. However, the total marital attitude score was not significantly associated with childbirth intention (η²=0.10, *p*=.787). These findings suggest that specific subdomains of marital attitudes, along with values regarding children, play more critical roles in shaping childbirth intentions than overall marital attitudes.

### Correlations between childbirth intention, marital attitude, values regarding children, and satisfaction factors

Table 3 presents correlations among childbirth intention, marital attitude, values regarding children, and satisfaction factors. Childbirth intention was moderately and positively correlated with values regarding children (r=.61, *p*<.001) and weakly but significantly correlated with economic satisfaction (r=.19, *p*=.003). No significant correlations were observed with total marital attitude (r=.02, *p*=.381) or nursing major satisfaction (r=.10, *p*=.078). Values regarding children were positively correlated with marital attitude (r=.15, *p*=.019) and with economic satisfaction (r=.18, *p*=.004). No multicollinearity was detected among these variables, as correlation coefficients were <.70.

### Moderating effect of values regarding children on the relationship between marital attitude and childbirth intention

**Figure 1.**
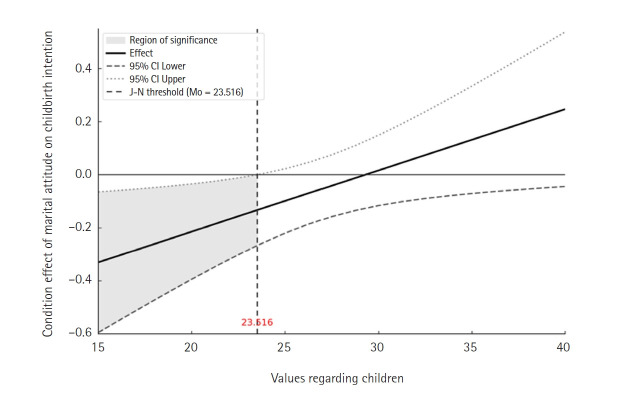
The conditional effect of marital attitude on fertility intention across levels of values regarding children. The vertical line indicates the Johnson–Neyman threshold at a score of 23.516 for values regarding children. Below this value (shaded region), marital attitude has a statistically significant negative effect on fertility intention. Above this threshold, the effect becomes nonsignificant as the 95% confidence interval (CI) includes zero. This suggests that negative marital attitudes reduce fertility intention primarily among individuals with low values regarding children.

Table 4 presents the hierarchical regression results. The interaction term between marital attitude and values regarding children was statistically significant (B=0.02, *p*=.037), indicating that values regarding children moderated the relationship between marital attitude and childbirth intention. The bootstrapped confidence interval (CI) for the interaction effect was significant (bootstrapped lower limit of the CI, 0.00; bootstrapped upper limit of the CI, 0.04), supporting the robustness of this finding. Johnson–Neyman analysis showed that when child-value scores were below 23.52, marital attitude had a significant negative effect on childbirth intention. However, at scores of 23.52 or higher, this effect was no longer significant. This indicates that a negative marital attitude exerted greater influence on childbirth intention among students with lower values regarding children (Figure 1, Table 5). Figure 1 was generated using an Excel macro for Johnson–Neyman 95% CI graphs developed by Lee [20]. Simple slope analysis further clarified the interaction. Among participants with low child values (mean – 1 SD, 21.19), marital attitude significantly predicted lower childbirth intention (B=–0.19, *p*=.029). In contrast, among those with moderate (mean, 26.31; *p*=.235) or high (mean + 1 SD, 31.43; *p*=.493) values regarding children, marital attitude was not significantly associated with childbirth intention. These findings suggest that negative marital attitudes reduce childbirth intention primarily among students who place relatively low value on having children. In this group, unfavorable marital attitudes appear to act as a stronger barrier to forming childbirth intentions.

## Discussion

This study investigated the factors influencing the intention to have children among nursing students and tested whether values regarding children moderate the relationship between marital attitude and childbirth intention. The PROCESS Model 1 analysis demonstrated that values regarding children significantly moderated this relationship. The Johnson–Neyman analysis further revealed that marital attitude had a significant negative effect on childbirth intention only when values regarding children were below 23.52. This finding aligns with the modified theory of planned behavior, which posits that intrinsic motivations, such as personal values, can buffer the behavioral influence of extrinsic attitudes [21,22]. In this context, values regarding children function as a psychological protective factor, mitigating the impact of negative marital attitudes on childbirth intention. Students with low values regarding children and skeptical views of marriage exhibited lower childbirth intentions, whereas those with high values maintained relatively stable intentions to have children regardless of marital attitudes. These results indicate that inner values actively moderate the influence of external attitudes on behavioral intentions, beyond the effects of background characteristics [17,21]. By empirically demonstrating this moderating effect, the present study extends previous findings that identified child values as predictors of childbirth intention among nursing and university students [6,8]. Its significance lies in showing how intrinsic value systems shape the extent to which extrinsic social attitudes influence reproductive decision-making.

Even after controlling satisfaction with nursing major, economic satisfaction, and sex, values regarding children consistently moderated the relationship between marital attitude and childbirth intention. This suggests that, regardless of students’ perceptions of their academic path or financial situation, low child-related values enable the negative influence of marital attitudes to persist. These findings imply that core psychological values about children exert a deeper and more stable influence than contextual satisfaction factors [21,22]. Consistent with this, one study reported that reproductive responsibility and child values explained childbirth intention more effectively than external situational factors [8], while another found that psychological attitudes toward marriage and family had a stronger impact than demographic characteristics among nursing students [17].

The present study contributes to the literature by providing empirical support for value-based educational approaches over knowledge-centered methods in promoting childbirth intention among nursing students. Specifically, students with lower child-related values were more vulnerable to the negative effects of passive or skeptical marital attitudes, underscoring the need for targeted interventions that foster constructive perspectives on parenting, family life, and reproductive planning.

As future maternal and child health professionals, nursing students require structured opportunities within their curriculum to explore and strengthen values related to childbearing. One practical educational strategy is the integration of value-reflection content into existing nursing courses. Elective subjects or seminar-based modules addressing parenting, family roles, and reproductive decision-making could encourage students to critically examine their values and form coherent perspectives on childbirth and life planning [4,6]. Such interventions may be especially valuable for students ambivalent about marriage or who hold low child-related values, as this study shows they are more susceptible to reduced childbirth intention. These findings are consistent with earlier studies highlighting child and family values as strong predictors of fertility intention among nursing students [4,8], as well as research in broader university populations [11]. Moreover, international evidence from systematic reviews confirms that child-related values exert a more consistent influence on reproductive intentions than external conditions such as economic satisfaction or educational attainment [7,21]. Collectively, these findings emphasize the importance of designing educational interventions that cultivate core psychological values alongside addressing contextual influences [4,21].

Values regarding children not only demonstrated the strongest positive correlation with childbirth intention (r=.61, *p*<.001) but also emerged as the most significant predictor in the regression analysis. This supports previous findings that positive child values are central determinants of childbirth intention among Korean female university students [8,21]. Furthermore, the moderating role of child values observed here echoes prior research showing that fertility decisions are shaped by how young women in Korea and Japan perceive marriage, gender roles, and family life across cultural contexts [21]. Importantly, this study found that students with lower child values were more vulnerable to negative marital attitudes, suggesting that value-based interventions may be especially critical for groups at risk of reduced childbirth intention.

Overall, these findings highlight the importance of curriculum development that incorporates structured reflection on parenting and family values within both clinical and theoretical nursing education [4]. Such approaches may be particularly effective for students with low child-related values, who were identified in this study as more vulnerable to reduced childbirth intention [8]. International reviews similarly stress that internal value systems and long-term life planning are essential components of fertility-related education, particularly for populations with low reproductive motivation [7,21].

Regarding the marriage attitude subfactor passive attitude, which showed a strong negative correlation (r=–.45, *p*<.001) with childbirth intention and emerged as a significant negative predictor in the regression model, this finding is consistent with prior research showing that students with skeptical or passive views of marriage tend to express lower willingness to have children, a construct closely tied to childbirth intention [5,16]. Conservative attitude demonstrated a positive correlation with childbirth intention and was a significant positive predictor, consistent with studies suggesting that traditional family values and marital stability are linked with stronger reproductive motivation [4,8]. An active attitude was also positively correlated with childbirth intention, although its significance in the regression model was marginal. This implies that perceiving marriage as a positive and active life choice may encourage childbirth intention. However, in this study, the effect was attenuated by moderating factors such as values regarding children, supporting earlier findings that the influence of marital attitudes on fertility intentions may vary depending on contextual variables such as parenting values or family-related priorities [8,21]. By contrast, romantic, exclusive, and instrumental attitudes were not significant predictors in the regression analysis. This suggests that practical and psychosocial aspects of marriage—reflected in passive, conservative, and active attitudes—may play a more decisive role in shaping childbirth intention than emotional or idealized views of marriage. These results are consistent with previous studies indicating that responsible, reality-based marital attitudes are more closely linked to fertility intention than romantic or symbolic perspectives [5,17].

Based on these findings, programs designed to increase childbirth intention—for example, initiatives that help nursing students explore strategies to balance future family responsibilities with professional roles [7] and efforts to strengthen values regarding children [8,21]—may be beneficial. In addition, workshops featuring senior nurses who have successfully combined professional practice with marriage and family life could provide practical strategies and real-world examples. Such workshops may be particularly valuable for students with passive marital attitudes, offering them opportunities to reflect on ways to integrate career development with family planning [5,17].

Considering that greater satisfaction with both nursing major and economic situation was associated with higher childbirth intention [11,17], enhancing students’ academic and career satisfaction may indirectly contribute to raising childbirth intentions. Examples include improving the quality of clinical training and expanding career counseling opportunities [4]. Furthermore, educational content that holistically addresses pregnancy, childbirth, bioethics, and family values may help nursing students align professional knowledge with personal beliefs. Such value-integrated education could serve as a foundation for students of childbearing age to cultivate positive and realistic attitudes toward marriage and future parenthood [4,21].

This study has several limitations. First, its generalizability may be restricted, as participants were drawn exclusively from nursing programs, and findings may not extend to students in other academic disciplines or the broader young adult population. Second, the study did not distinguish between students who had completed versus those currently enrolled in women’s health or pediatric nursing practicum, and this variation in clinical exposure may have influenced attitudes and childbirth intentions. Third, the cross-sectional design prevents assessment of temporal changes or causal relationships between marital attitudes, values regarding children, and childbirth intention. Future research should incorporate longitudinal designs and qualitative methods, such as in-depth interviews conducted before and after relevant coursework or clinical practicum, to examine how educational experiences shape students’ values and reproductive intentions over time.

In conclusion, this study demonstrated that nursing students’ values regarding children were the strongest determinant of childbirth intention and significantly buffered the negative influence of unfavorable marital attitudes. After controlling satisfaction with nursing major, economic satisfaction, and sex, results showed that students with low child values reported significantly reduced childbirth intention when holding negative marital views, whereas those with high child values were protected from such effects. These findings suggest that interventions aimed at clarifying and reinforcing child-related values, alongside fostering positive marital attitudes, may enhance childbirth intention among nursing students of childbearing age.

## Figures and Tables

**Table 1.** Childbirth intention according to general characteristics (N=205)

Variable	Categories	Childbirth intention		
		n (%)	Mean±SD	t or F (*p*)
Sex	Male	43 (21.0)	32.23±5.36	4.37 (<.001)
	Female	162 (79.0)	28.07±6.24	
Age (year)	<25^a^	156 (76.1)	28.28±6.48	4.37 (.014) b>a,c^†^
	25–30^b^	43 (21.0)	31.42±5.05	
	≥31^c^	6 (2.9)	28.50±5.36	
Year in the program (year)	3rd	77 (37.6)	27.88±6.52	–1.88 (.061)
	4rd	128 (62.4)	29.58±6.08	
Nursing major satisfaction	Satisfied^a^	120 (58.5)	29.47±6.32	7.36 (<.001) a, b>c^†^
	Moderate^b^	70 (34.1)	29.29±5.87	
	Dissatisfied^c^	15 (7.3)	23.13±6.29	
Economic satisfaction	Satisfied^a^	80 (39.0)	30.43±6.61	4.26 (.015) a>c^†^
	Moderate^b^	96 (46.8)	28.29±5.74	
	Dissatisfied^c^	29 (14.2)	27.00±6.44	
Religion	Yes	116 (56.6)	29.76±6.16	1.63 (.185)
	None	89 (43.4)	27.84±6.38	
Birth order	First	85 (41.5)	28.93±6.30	1.66 (.178)
	Second	86 (42.0)	28.42±6.49	
	Third	29 (14.1)	29.55±5.54	
	≥Fourth	5 (2.4)	34.60±4.83	
Parents’ marital status	Married	165 (80.5)	28.93±6.44	0.70 (.556)
	Separated	21 (10.2)	27.71±5.79	
	Widowed	11 (5.4)	29.82±5.44	
	Remarried	8 (3.9)	31.25±6.29	
Living with parents	Yes	149 (72.7)	29.27±6.47	1.22 (.225)
	No	56 (27.3)	28.07±5.73	

SD: Standard deviation.

† Post-hoc test analyzed using the Scheffé method.

**Table 2.** Childbirth intention, marital attitude, values regarding children among nursing students (N=205)

Variable	Mean±SD	Possible range	Point-average, mean±SD	Range	η² (*p*)
Childbirth intention	28.94±6.29	11–55	2.63±0.57	13–43	
Marital attitude (total)	69.60±6.01	20–100	3.48±0.30	54–84	.10 (.787)
Romantic	21.15±2.26	5–25	4.23±0.45	14–25	.11 (.019)
Passive	19.35±4.01	6–30	3.23±0.67	8–30	.27 (<.001)
Conservative	7.86±2.19	3–15	2.62±0.73	3–15	.12 (.007)
Exclusive	7.32±1.47	2–10	3.66±0.73	2–10	.12 (.001)
Active	6.25±1.77	2–10	3.13±0.88	2–10	.25 (<.001)
Instrumental	7.68±1.60	2–10	3.84±0.80	2–10	.08 (.043)
Values regarding children	26.31±5.12	8–40	3.29±0.64	15–40	.45 (<.001)

η²: Partial eta squared.

**Table 3.** Relationships among childbirth intention, marital attitude, values regarding children, and satisfaction factors (N=205)

Variable	r (*p*)			
	Childbirth intention	Marital attitude	Values regarding children	Nursing major satisfaction
Childbirth intention	1			
Marital attitude	.02 (.381)	1		
Values regarding children	.61 (<.001)	.15 (.019)	1	
Nursing major satisfaction (satisfied=1)	.10 (.078)	–0.08 (.137)	.08 (.137)	1
Economic satisfaction (satisfied=1)	.19 (.003)	–0.01 (.440)	.18 (.004)	.06 (.180)

**Table 4.** Moderating effect of values regarding children on the relationship between marital attitude and childbirth intention (N=205)

Moderating variable	Variable	B	SE	t (*p*)	95% CI
Values regarding children (Z)	(Constant)	58.62	19.86	2.99 (.003)	19.98 to 96.57
	Marital attitude (X)	–0.68	0.29	–2.40 (.017)	–1.24 to –0.10
	X × Z (interaction)	0.02	0.01	2.25 (.026)	0.00 to 0.04
	Nursing major satisfaction (satisfied=1) (covariate)	0.82	0.72	1.15 (.251)	–0.59 to 2.24
	Economic satisfaction (satisfied=1) (covariate)	1.04	0.73	1.43 (.154)	–0.39 to 2.47
	Sex (covariate)	–1.06	0.91	–1.16 (.247)	–2.86 to 0.74
	*R^2^* change of interaction item	*R^2^* (adjusted R^2^)		F	*p*
		.401 (.383)		22.10	<.001

CI, Confidence interval; SE, standard error. Durbin-Watson=2.148. All interaction terms had variance inflation factors ≤10, indicating no serious multicollinearity. The conditional effects were estimated at the mean and ±1 standard deviation of the moderator.

**Table 5.** Conditional effects of marital attitude on childbirth intention based on values regarding children (N=205)

Level of moderator^†^	BootB	BootSE	*p*	Bootstrapped 95% CI
Low (–1 SD = 21.19)	–0.19	0.08	.029	–0.35 to –0.03
Moderate (Mean = 26.31)	–0.07	0.06	.235	–0.19 to 0.05
High (+1 SD = 31.43)	–0.05	0.08	.493	–0.10 to 0.20

CI: Confidence interval; Boot B: bootstrapped indirect effect; BootSE: bootstrapped standard error; SD: standard deviation. Bootstrapping based on 5,000 samples. Effects were considered statistically significant if the 95% CI did not include zero.

† Level of values regarding children.
